# Coronary computed tomographic angiography for patients with low-to-intermediate risk chest pain: A systematic review and meta-analysis

**DOI:** 10.18632/oncotarget.13782

**Published:** 2016-12-02

**Authors:** Yu Chen, Yuqi Fan, Zhaofang Yin, Huili Zhang, Yang Zhang, Zhihua Han, Changqian Wang

**Affiliations:** ^1^ Department of Cardiology, Shanghai Ninth Peoples Hospital, Shanghai Jiao Tong University School of Medicine, Shanghai, China

**Keywords:** coronary computed tomographic angiography, chest pain, coronary heart disease, functional test, meta-analysis

## Abstract

Coronary computed tomographic angiography (CCTA) can image the coronary vasculature rapidly and detect the presence and severity of luminal stenosis accurately. However, whether CCTA based care strategy could gain more benefits than conventional strategy with functional tests for patients with low-to-intermediate risk chest pain remains unknown. In this study we performed a meta-analysis to compare the clinical efficacy of CCTA versus conventional strategy. Eight randomized controlled trials with 14749 patients were finally included in this review after database searching. Compared with conventional strategy, CCTA significantly increased the rates of invasive coronary angiography (RR 1.44; 95% CI 1.28 to 1.63) and revascularization (RR 1.94; 95% CI 1.65 to 2.29), but did not change the rates of major adverse cardiovascular events (RR 1.10; 95% CI 0.92 to 1.30), death (RR 0.95; 95% CI 0.64 to 1.40) and hospital readmission (RR 0.96; 95% CI 0.66 to 1.40). Consequently, compared with conventional strategy, CCTA seemed not to improve clinical outcomes for patients with low-to-intermediate risk chest pain.

## INTRODUCTION

Low-to-intermediate risk chest pain is a common clinical issue in emergency departments (ED) [1]. Several noninvasive tests, including coronary computed tomographic angiography (CCTA), myocardial perfusion imaging (MPI), stress electrocardiogram (ECG), and stress echocardiography, have been performed for the patients with suspected coronary artery disease (CAD), however, there has been little consensus about which testing strategy is optimal [2–4].

Compared to functional tests, CCTA can image the coronary vasculature rapidly and detect the presence and severity of luminal stenosis accurately [5,6]. However, whether CCTA based care strategy could gain more clinical benefits than conventional care strategy with functional tests for patients with low-to-intermediate risk chest pain remains unknown.

El-Hayek et al. carried out a meta-analysis to show CCTA based care strategy reduced the risk of future adverse cardiovascular events and subsequent ED visits among patients with low-to-intermediate risk chest pain [7]. However, their conclusions were inherently unreliable, because there was high heterogeneity among studies included in the meta-analysis. Furthermore, some new related trials were performed recent years, but their conclusions were conflicting [8–11]. As a result, we carried out a meta-analysis to quantify the available clinical evidences on efficacy of CCTA versus conventional care strategy in patients with low-intermediate risk chest pain.

## RESULTS

### Included studies

**Figure 1:**
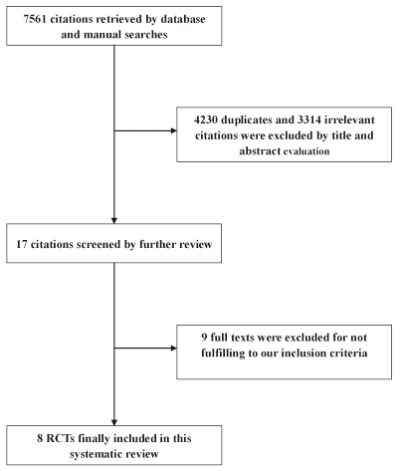
Flow chart of study selection

**Figure 2:**
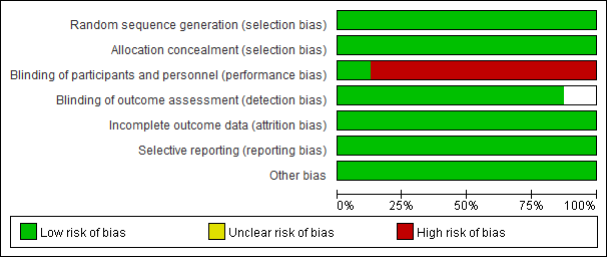
Risk of bias graph

The selection process of studies is shown in Figure 1. Finally, eight randomized controlled trials (RCTs) with 14749 patients were included in the review [8–15]. The κ value was 0.76, indicating a satisficing inter-observer agreement for study selection. The characteristics of eligible RCTs are detailed given in Table 1. The methodological qualities were assessed (Figure 2).

**Table 1:** Main characteristics of the included RCTs

RCTs	Age (mean)	Male	HTN	DM	Dyslipidemia	Smoking	Family history	Follow up	Previous CAD	Functional test type
Goldstein 2007 [12]	50	50%	39%	10%	36%	18%	42%	6m	0%	MPI
Goldstein 2011 [13]	50	46%	37%	7%	33%	22%	30%	6m	0%	MPI
Hoffman 2012 [14]	54	53%	54%	17%	46%	50%	27%	1m	0%	MPI/sECG/sUCG
Linde 2013 [15]	56	57%	42%	11%	38%	64%	25%	4m	14%	MPI/sECG
Hamilton-Craig 2014[11]	52	58%	31%	7%	25%	23%	33%	12m	0%	sECG
Hollander 2015[9]	49	46%	51%	16%	28%	32%	29%	12m	NA	MPI/sECG/sUCG
Douglas 2015[8]	61	47%	65%	21%	68%	16%	32%	25m	0%	MPI/sECG/sUCG
Levsky 2015[10]	57	37%	72%	32%	51%	15%	NA	12m	0%	MPI

Abbreviations: RCT: randomized controlled trial; CAD: coronary atherosclerosis heart disease; HTN: hypertension; DM: diabetes mellitus;MPI: myocardial perfusion imaging; sECG: stress electrocardiograph; sUCG: stress echocardiography; NA: not applicable.

### Clinical endpoints

**Figure 3:**
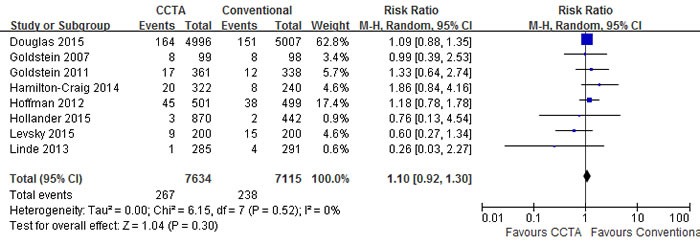
Comparisons of CCTA versus conventional strategy on MACE

**Figure 4:**
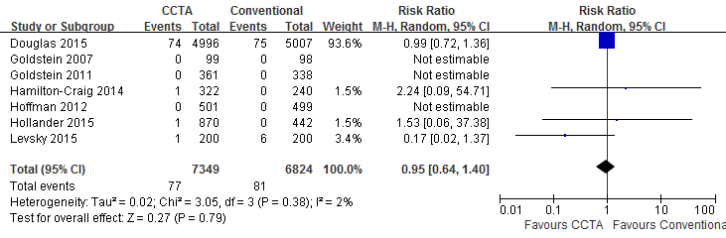
Comparisons of CCTA versus conventional strategy on death

**Figure 5:**
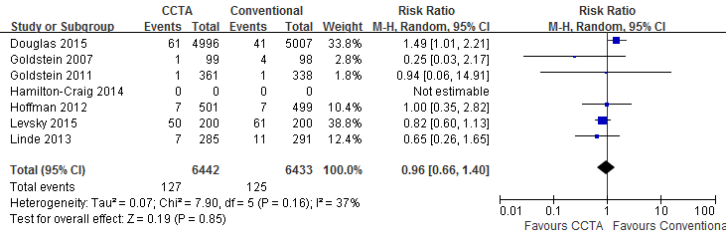
Comparisons of CCTA versus conventional strategy on hospital readmission

**Figure 6:**
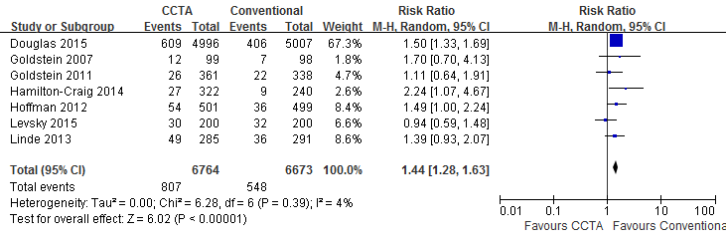
Comparisons of CCTA versus conventional strategy on ICA

**Figure 7:**
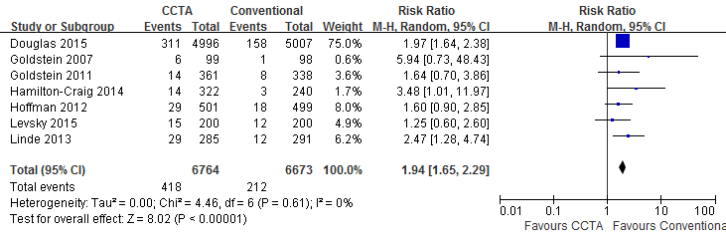
Comparisons of CCTA versus conventional strategy on revascularization

A pooled risk ratio (RR) with 95% confidence interval (CI) was used to measure the overall treatment effect for each endpoint. Compared with conventional care strategy, CCTA did not reduce the risks of major adverse cardiovascular events (MACE) (RR 1.10; 95% CI 0.92 to 1.30; I2 = 0%; p = 0.30), death (RR 0.95; 95% CI 0.64 to 1.40; I2 = 2%; p = 0.79) and hospital readmission (RR 0.96; 95% CI 0.66 to 1.40; I2 = 37%; p = 0.85) (Figures 3, 4, 5). However, CCTA significantly increased the rates of invasive coronary angiography (ICA) (RR 1.44; 95% CI 1.28 to 1.63; I2 = 4%; p < 0.001) and revascularization (RR 1.94; 95% CI 1.65 to 2.29; I2 = 0%; p < 0.001) (Figures 6, 7).

### Subgroup analyses

Subgroup analyses were performed between short-term (no more than 6 month) and long-term (no less than 12 month) follow-up (Table 2). Most clinical endpoints did not change significantly between the two subgroups except ICA. Specially, CCTA was associated with higher ICA rate in 6 months (RR 1.38; 95% CI 1.09 to 1.76; I2 = 0%; p = 0.008), while it did not increase ICA rate significantly during long-term follow-up (RR 1.40; 95% CI 0.96 to 2.03; I2 = 61%; p = 0.08).

**Table 2:** Subgroup analyses

	Short-term Follow-up	Long-term Follow-up
MACE	1.41 (0.82, 1.59)	1.06 (0.74, 1.53)
Death	zero events	0.95 (0.64, 1.40)
Hospital readmission	0.72 (0.38, 1.36)	1.09 (0.60, 1.98)
ICA	1.38 (1.09, 1.76)	1.40 (0.96, 2.03)
Revascularization	1.94 (1.33, 2.84)	1.91 (1.45, 2.52)

### Heterogeneity assessment and publication bias

**Figure 8:**
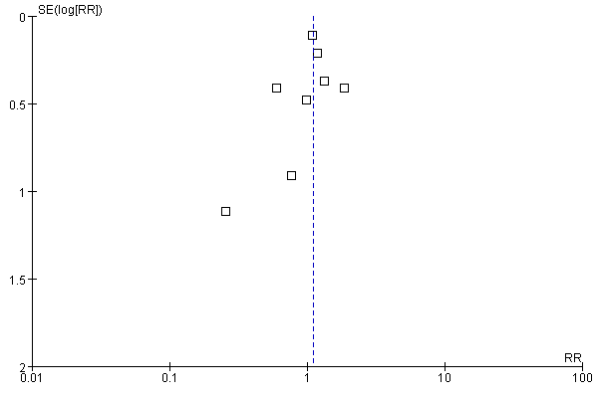
Funnel plots of MACE for CCTA versus conventional strategy

The I2 values of MACE, death, ICA and revascularization were equal or approximate to zero, implying no heterogeneity for these endpoints. The I2 value of hospital readmission was 37%, indicating a moderate heterogeneity. To evaluate the effects of heterogeneities among different studies on the clinical endpoints, we performed a sensitivity analysis by removing one study once. After excluding anyone study once, all the results of these endpoints did not significantly change. To detect the small-study effect, the results between the random-effect model and fixed-effect model were compared accounting to the recommendation of Cochrane Collaboration [16], and found that their results were similar. A mild publication bias might exist, as a mild asymmetry in the funnel plot of MACE (Figure 8).

## DISCUSSION

Compared with conventional strategy, CCTA increased the rate of ICA, which may be because CCTA could increase the identification of coronary atherosclerosis, subsequently warranting to be validated by ICA. Furthermore, CCTA was associated with the higher rate of revascularization, which was closely related to the increased ICA rate. However, CCTA did not reduce the rates of MACE, death and hospital readmission.

Higher revascularization rate could lead to less MACE and hospital readmission ideally. However, our results were not consistent with it. Compared with conventional strategy, CCTA did not reduce the rates of MACE, death and hospital readmission significantly. The possible reasons might be as follows. For one reason, the included patients were with low-to-intermediate risk chest pain and most of their coronary arteries were normal or mild abnormal. The low incidence of cardiac events of these patients requires greater size of study population. Boden et al. revealed that coronary revascularization was not related to improved clinical outcomes in stable CAD patients, which might be the same reason [17]. In contrast to stable CAD, Fox et al. demonstrated that revascularization was associated with improved outcomes in patients with acute coronary syndromes [18]. For another reason, the follow-up duration of most included trials were not more than one year, the potential benefits of revascularization might have not been emerged, which need longer-term observe in future.

Our findings were different from El-Hayek et al's to a large extent. They demonstrated that CCTA reduced the risks of adverse cardiovascular events without changing the rates of ICA and revascularization of patients with low-to-intermediate risk chest pain [7]. In their study, there were only four RCTs and three non-RCTs performed from 2007 to 2011. The results of the four RCTs showed that CCTA increased the rates of ICA and revascularization, but could not change the adverse cardiovascular events risk. However, when combined with three non-RCTs, all the above results reversed. In view of the high heterogeneity between the two types of studies, it is not reasonable to combine RCTs with non-RCTs. Thus their conclusions need to be treated with caution.

We totally included eight RCTs performed from 2007 to 2015. All of the endpoints did not show significantly statistical heterogeneity except a moderate heterogeneity of ICA. To evaluate the effects of the statistical heterogeneity of ICA and possible clinical heterogeneity among different studies, we performed a sensitivity analysis and did not find significant influence caused by heterogeneity. Furthermore, we performed subgroup analyses and found all the clinical endpoints did not change significantly between the subgroups except for a mild increase of ICA in short-term subgroup. Besides, small-study effects were not found in our study. As a result, our conclusions were more credible.

In conclusion, our review demonstrates that compared with conventional strategy, CCTA based strategy significantly increased the rates of ICA and revascularization, but did not change the rates of MACE, death and hospital readmission. Due to the relatively short follow-up duration, some longer follow-up RCTs are warrant in future.

## materials and METHODS

### Search strategy

This meta-analysis was written according to the PRISMA [19] and Cochrane Collaboration guidelines [16]. We searched PUBMED, EMBASE, Web of Knowledge, and the Cochrane Central Register of Controlled Trials for relevant RCTs with the following strategies. Keywords relevant to coronary computed tomography (“coronary computed tomography angiography” or “coronary computed tomography” or “CCTA” [Title/Abstract]) were combined with Clinical Trial[ptyp]. Besides, we performed an extensive manual search. Relevant literatures were referred, and websites for recent trials were searched.

### Inclusion criteria and Outcome measures

The following RCTs were included: (1) RCTs comparing CCTA versus conventional care; (2) patients with suspected CAD; (3) follow-up ≥30 days. RCTs were excluded: (1) ongoing studies, (2) duplicate reports, (3) incomplete follow-up. Two investigators (C. Y, and H. ZH) independently reviewed the titles, abstracts, or full-texts to determine whether the studies met the selection criteria. Conflicts between them were consulted by a third investigator (W. CQ). The incidence of MACE, defined as composite events of unstable angina pectoris (UAP), MI and cardiovascular death, was chosen as the primary endpoint. The secondary endpoints were the incidences of hospital readmission, death, ICA, or revascularization.

### Data selection and quality assessment

The following data of each RCT were extracted: author, publication year, number of patients, gender, age, intervention strategy, clinical outcomes, concomitant medication, main past medical history and follow-up. All data were independently extracted by two investigators (Z. HL and Z. Y). Disagreements were consulted with a third reviewer (W. CQ). We assessed the qualities of included RCTs by the risks of biases according to the Cochrane Collaboration [16].

### Statistical analysis

The meta-analyses were carried out with Review Manager 5.1. Two-tailed p-value of less than 0.05 was defined statistically significant. Agreement for study selection was assessed by κ statistic. A RR with 95% CI was used for measuring treatment effect of each study. The pooled RR with 95% CI was estimated by a random effect model (DerSimonian-Laird) for measuring the overall treatment effect. Subgroup analyses were performed between short-term (no more than 6 month) and long-term (no less than 12 month) follow-up. Heterogeneity between RCTs was assessed by I2 test. I2 value of zero indicated there was no heterogeneity. I2 values < 25%, 25% < I2 value < 50% , and > 50% represented low, moderate, and high degree of heterogeneities [20]. To explore the effects of heterogeneity, sensitivity analysis was performed by removing one trial each time. The publication bias was evaluated by a funnel plot [21].
